# Supplementary material for: From sequence to enzyme mechanism using multi-label machine learning
Source: BMC Bioinformatics. 2014 May 19;15:150. doi: 10.1186/1471-2105-15-150 (PMC4229970; doi:10.1186/1471-2105-15-150)
Supplement: Additional file 2 — Java code of ml2db. Additional file ml2db_code.tar.gz contains the Java source code to run the multi-label machine learning experiments and save the results to database. The code’s Javadoc is included. [file 1471-2105-15-150-S2.zip › additional file 2/ml2db/ml2db/doc/mulan/data/class-use/MultiLabelInstances.html]

Uses of Class mulan.data.MultiLabelInstances


JavaScript is disabled on your browser.


- Overview
- Package
- Class
- Use
- Tree
- Deprecated
- Index
- Help

- Prev
- Next

- Frames
- No Frames

- All Classes

## Uses of Class mulan.data.MultiLabelInstances

- Packages that use MultiLabelInstances

  | Package | Description |
  | --- | --- |
  |  |  |
  | --- | --- |
  | mulan.classifier |  |
  | mulan.classifier.meta |  |
  | mulan.classifier.meta.thresholding |  |
  | mulan.classifier.neural |  |
  | mulan.classifier.transformation |  |
  | mulan.data |  |
  | mulan.dimensionalityReduction |  |
  | mulan.evaluation |  |
  | mulan.evaluation.loss |  |
  | mulan.evaluation.measure |  |
  | mulan.transformations |  |
  | mulan.transformations.multiclass |  |
- - ### Uses of MultiLabelInstances in mulan.classifier

    Methods in mulan.classifier with parameters of type MultiLabelInstances

    | Modifier and Type | Method and Description |
    | --- | --- |
    |  |  |
    | --- | --- |
    | `void` | MultiLabelLearnerBase.`build(MultiLabelInstances trainingSet)` |
    | `void` | MultiLabelLearner.`build(MultiLabelInstances instances)` Builds the learner model from specified `MultiLabelInstances` data. |
  - ### Uses of MultiLabelInstances in mulan.classifier.meta

    Methods in mulan.classifier.meta that return MultiLabelInstances

    | Modifier and Type | Method and Description |
    | --- | --- |
    |  |  |
    | --- | --- |
    | `MultiLabelInstances` | HierarchyBuilder.`buildHierarchy(MultiLabelInstances mlData)` Builds a hierarhical multi-label dataset. |
    | `MultiLabelInstances` | HierarchyBuilder.`buildHierarchyAndSaveFiles(MultiLabelInstances mlData, java.lang.String arffName, java.lang.String xmlName)` Builds the hierarchy and constructs auxiliary files |
    | `static MultiLabelInstances` | HierarchyBuilder.`createHierarchicalDataset(MultiLabelInstances mlData, LabelsMetaData metaData)` Creates the hierarchical dataset according to the original multilabel instances object and the constructed label hierarchy |

    Methods in mulan.classifier.meta with parameters of type MultiLabelInstances

    | Modifier and Type | Method and Description |
    | --- | --- |
    |  |  |
    | --- | --- |
    | `MultiLabelInstances` | HierarchyBuilder.`buildHierarchy(MultiLabelInstances mlData)` Builds a hierarhical multi-label dataset. |
    | `MultiLabelInstances` | HierarchyBuilder.`buildHierarchyAndSaveFiles(MultiLabelInstances mlData, java.lang.String arffName, java.lang.String xmlName)` Builds the hierarchy and constructs auxiliary files |
    | `void` | ClusteringBased.`buildInternal(MultiLabelInstances trainData)` |
    | `LabelsMetaData` | HierarchyBuilder.`buildLabelHierarchy(MultiLabelInstances mlData)` Builds a hierarhy of labels on top of the labels of a flat multi-label dataset, by recursively partitioning the labels into a specified number of partitions. |
    | `static MultiLabelInstances` | HierarchyBuilder.`createHierarchicalDataset(MultiLabelInstances mlData, LabelsMetaData metaData)` Creates the hierarchical dataset according to the original multilabel instances object and the constructed label hierarchy |
    | `void` | RAkELd.`updateClassifier(MultiLabelInstances mlTrainData, int model)` Updates the current ensemble by training a specific classifier |
  - ### Uses of MultiLabelInstances in mulan.classifier.meta.thresholding

    Methods in mulan.classifier.meta.thresholding with parameters of type MultiLabelInstances

    | Modifier and Type | Method and Description |
    | --- | --- |
    |  |  |
    | --- | --- |
    | `weka.core.Instances` | ThresholdPrediction.`transformData(MultiLabelInstances trainingData)` |
  - ### Uses of MultiLabelInstances in mulan.classifier.neural

    Methods in mulan.classifier.neural with parameters of type MultiLabelInstances

    | Modifier and Type | Method and Description |
    | --- | --- |
    |  |  |
    | --- | --- |
    | `static java.util.List<DataPair>` | DataPair.`createDataPairs(MultiLabelInstances mlDataSet, boolean bipolarOutput)` Creates a `DataPair` representation for each `Instance` contained in `MultiLabelInstances` data set. |

    Constructors in mulan.classifier.neural with parameters of type MultiLabelInstances

    Constructor and Description

    `NormalizationFilter(MultiLabelInstances mlData,
    boolean performNormalization)`

    Creates a new instance of `NormalizationFilter` class for given data set.

    `NormalizationFilter(MultiLabelInstances mlData,
    boolean performNormalization,
    double minValue,
    double maxValue)`

    Creates a new instance of `NormalizationFilter` class for given data set.
  - ### Uses of MultiLabelInstances in mulan.classifier.transformation

    Methods in mulan.classifier.transformation with parameters of type MultiLabelInstances

    | Modifier and Type | Method and Description |
    | --- | --- |
    |  |  |
    | --- | --- |
    | `void` | MultiLabelStacking.`buildBaseLevel(MultiLabelInstances trainingSet)` Builds the base-level classifiers. |
    | `void` | MultiLabelStacking.`buildBaseLevelKNN(MultiLabelInstances trainingSet)` Used only in case of a kNN base classifier. |
    | `void` | IncludeLabelsClassifier.`buildInternal(MultiLabelInstances mlData)` |
    | `void` | MultiLabelStacking.`initializeMetaLevel(MultiLabelInstances dataSet, weka.classifiers.Classifier metaClassifier, boolean includeAttrs, double metaPercentage, weka.attributeSelection.ASEvaluation eval)` Initializes all the parameters used in the meta-level. |
  - ### Uses of MultiLabelInstances in mulan.data

    Methods in mulan.data that return MultiLabelInstances

    | Modifier and Type | Method and Description |
    | --- | --- |
    |  |  |
    | --- | --- |
    | `MultiLabelInstances` | MultiLabelInstances.`clone()` Returns a deep copy of the `MultiLabelInstances` instance. |
    | `MultiLabelInstances` | MultiLabelInstances.`reintegrateModifiedDataSet(weka.core.Instances modifiedDataSet)` If `Instances` data set are retrieved from `MultiLabelInstances` and post-processed, modified by custom code, it can be again reintegrated into `MultiLabelInstances` if needed. |
    | `MultiLabelInstances[]` | LabelPowersetStratification.`stratify(MultiLabelInstances data, int folds)` |
    | `MultiLabelInstances[]` | IterativeStratification.`stratify(MultiLabelInstances data, int folds)` |
    | `MultiLabelInstances[]` | Stratification.`stratify(MultiLabelInstances data, int folds)` Creates a number of folds via stratified sampling |

    Methods in mulan.data with parameters of type MultiLabelInstances

    | Modifier and Type | Method and Description |
    | --- | --- |
    |  |  |
    | --- | --- |
    | `double[][]` | Statistics.`calculateCoocurrence(MultiLabelInstances mdata)` This method calculates and prints a matrix with the coocurrences of   pairs of labels |
    | `LabelsPair[]` | LabelPairsDependenceIdentifier.`calculateDependence(MultiLabelInstances mlInstances)` Calculates dependence level between each pair of labels in the given multilabel data set |
    | `LabelsPair[]` | UnconditionalChiSquareIdentifier.`calculateDependence(MultiLabelInstances mlInstances)` Calculates Chi Square values for each pair of labels. |
    | `LabelsPair[]` | ConditionalDependenceIdentifier.`calculateDependence(MultiLabelInstances mlInstances)` Calculates t-statistic value for each pair of labels. |
    | `double[][]` | Statistics.`calculatePhi(MultiLabelInstances dataSet)` Calculates phi correlation |
    | `void` | Statistics.`calculateStats(MultiLabelInstances mlData)` calculates various multilabel statistics, such as label cardinality,   label density and the set of distinct labels along with their frequency |
    | `int[][]` | GreedyLabelClustering.`determineClusters(MultiLabelInstances trainingSet)` Determines labels partitioning into dependent sets. |
    | `int[][]` | LabelClustering.`determineClusters(MultiLabelInstances trainingSet)` Returns a label set partitioning into clusters |
    | `MultiLabelInstances[]` | LabelPowersetStratification.`stratify(MultiLabelInstances data, int folds)` |
    | `MultiLabelInstances[]` | IterativeStratification.`stratify(MultiLabelInstances data, int folds)` |
    | `MultiLabelInstances[]` | Stratification.`stratify(MultiLabelInstances data, int folds)` Creates a number of folds via stratified sampling |
  - ### Uses of MultiLabelInstances in mulan.dimensionalityReduction

    Methods in mulan.dimensionalityReduction with parameters of type MultiLabelInstances

    | Modifier and Type | Method and Description |
    | --- | --- |
    |  |  |
    | --- | --- |
    | `int[]` | Ranker.`search(weka.attributeSelection.AttributeEvaluator attributeEval, MultiLabelInstances mlData)` Calls a specified `AttributeEvaluator` to evaluate each feature attribute of specified `MultiLabelInstances` data set, excluding labels. |

    Constructors in mulan.dimensionalityReduction with parameters of type MultiLabelInstances

    Constructor and Description

    `BinaryRelevanceAttributeEvaluator(weka.attributeSelection.ASEvaluation ase,
    MultiLabelInstances mlData,
    java.lang.String combapp,
    java.lang.String norm,
    java.lang.String mode)`

    `LabelPowersetAttributeEvaluator(weka.attributeSelection.ASEvaluation x,
    MultiLabelInstances mlData)`

    Constructor that uses an evaluator on a multi-label dataset

    `MultiClassAttributeEvaluator(weka.attributeSelection.ASEvaluation x,
    MultiClassTransformation dt,
    MultiLabelInstances mlData)`

    Constructor that uses an evaluator on a multi-label dataset using a transformation
  - ### Uses of MultiLabelInstances in mulan.evaluation

    Methods in mulan.evaluation with parameters of type MultiLabelInstances

    | Modifier and Type | Method and Description |
    | --- | --- |
    |  |  |
    | --- | --- |
    | `void` | Evaluator.`checkData(MultiLabelInstances data)` |
    | `MultipleEvaluation` | Evaluator.`crossValidate(MultiLabelLearner learner, MultiLabelInstances data, int someFolds)` Evaluates a `MultiLabelLearner` via cross-validation on given data set with defined number of folds and seed. |
    | `MultipleEvaluation` | Evaluator.`crossValidate(MultiLabelLearner learner, MultiLabelInstances data, java.util.List<Measure> measures, int someFolds)` Evaluates a `MultiLabelLearner` via cross-validation on given data set using given evaluation measures with defined number of folds and seed. |
    | `Evaluation` | Evaluator.`evaluate(MultiLabelLearner learner, MultiLabelInstances data)` Evaluates a `MultiLabelLearner` on given test data set. |
    | `Evaluation` | Evaluator.`evaluate(MultiLabelLearner learner, MultiLabelInstances data, java.util.List<Measure> measures)` Evaluates a `MultiLabelLearner` on given test data set using specified evaluation measures |

    Constructors in mulan.evaluation with parameters of type MultiLabelInstances

    Constructor and Description

    `Evaluation(java.util.List<Measure> someMeasures,
    MultiLabelInstances data)`

    Creates a new evaluation object by deep copying the measure objects that
    are given as parameters

    `MultipleEvaluation(Evaluation[] someEvaluations,
    MultiLabelInstances data)`

    Constructs a new object with given array of evaluations

    `MultipleEvaluation(MultiLabelInstances data)`

    Constructs a new object
  - ### Uses of MultiLabelInstances in mulan.evaluation.loss

    Constructors in mulan.evaluation.loss with parameters of type MultiLabelInstances

    Constructor and Description

    `HierarchicalLoss(MultiLabelInstances data)`

    Creates a new instance of this class
  - ### Uses of MultiLabelInstances in mulan.evaluation.measure

    Constructors in mulan.evaluation.measure with parameters of type MultiLabelInstances

    Constructor and Description

    `HierarchicalLoss(MultiLabelInstances data)`

    Creates an instance of this object based on the corresponding loss
    function
  - ### Uses of MultiLabelInstances in mulan.transformations

    Methods in mulan.transformations with parameters of type MultiLabelInstances

    | Modifier and Type | Method and Description |
    | --- | --- |
    |  |  |
    | --- | --- |
    | `static weka.core.Instances` | RemoveAllLabels.`transformInstances(MultiLabelInstances mlData)` |
    | `weka.core.Instances` | LabelPowersetTransformation.`transformInstances(MultiLabelInstances mlData)` |
    | `weka.core.Instances` | IncludeLabelsTransformation.`transformInstances(MultiLabelInstances mlData)` |

    Constructors in mulan.transformations with parameters of type MultiLabelInstances

    Constructor and Description

    `BinaryRelevanceTransformation(MultiLabelInstances data)`

    Constructor
  - ### Uses of MultiLabelInstances in mulan.transformations.multiclass

    Methods in mulan.transformations.multiclass with parameters of type MultiLabelInstances

    | Modifier and Type | Method and Description |
    | --- | --- |
    |  |  |
    | --- | --- |
    | `weka.core.Instances` | SelectBasedOnFrequency.`transformInstances(MultiLabelInstances mlData)` |
    | `weka.core.Instances` | MultiClassTransformationBase.`transformInstances(MultiLabelInstances mlData)` |
    | `weka.core.Instances` | MultiClassTransformation.`transformInstances(MultiLabelInstances dataSet)` Transforms a multi-label dataset to a multi-class single label dataset |

- Overview
- Package
- Class
- Use
- Tree
- Deprecated
- Index
- Help

- Prev
- Next

- Frames
- No Frames

- All Classes
